# Progressive Acquired Cervical Deformity With Myelopathy Managed by Occipitocervicothoracic Fusion: A Case Report

**DOI:** 10.7759/cureus.106603

**Published:** 2026-04-07

**Authors:** Kyle Molinari, Nicolas A Siegelman, Steven Leckie

**Affiliations:** 1 Orthopedic Surgery, Plymouth Bay Orthopedics, Plymouth, USA; 2 Osteopathic Medicine, Nova Southeastern University Dr. Kiran C. Patel College of Osteopathic Medicine, Clearwater, USA; 3 Orthopedic Surgery, Beth Israel Deaconess Medical Center, Plymouth, USA

**Keywords:** cervical disc degeneration, cervical kyphoscoliosis, cervical spinal stenosis, cervical spine deformity, degenerative cervical myelopathy, occipito-cervical fusion surgery, posterior cervical laminectomy and fusion, vertebral artery anomaly

## Abstract

Progressive cervical myelopathy associated with acquired cervical deformity is uncommon and presents substantial diagnostic and surgical challenges, particularly when the deformity is rigid and complicated by anomalous vascular anatomy. Severe focal stenosis at the apex of cervical kyphosis and segmental autofusion may increase the risk of neurologic injury during decompression and hinder deformity correction.

We report the case of a 55-year-old woman with progressive cervical myelopathy in the setting of an acquired cervical kyphoscoliosis. Although no definitive traumatic event was identified, remote trauma was suspected to have contributed to the development of the cervical deformity and subsequent stenosis. Imaging demonstrated severe focal stenosis from C4-6 at the kyphotic segment, spondylolisthesis at C3-4 and C4-5, autofusion through the C3-4 disc space and right facet joint, and marked deformity of the left C3 and C4 lateral masses. Additionally, atypical vertebral artery anatomy from C2 to C4 on the left increased the risk associated with posterior cervical instrumentation. The deformity was rigid and failed to correct with traction.

Given progressive neurologic decline and severe cord compression, the patient underwent posterior decompression and stabilization with C3-7 laminectomy and occiput-T2 fusion. Instrumentation included an occipital plate, C2 pars screws, subaxial lateral mass screws where feasible, and T1-T2 pedicle screws (DePuy-Synthes Symphony, Johnson & Johnson MedTech, Warsaw, USA). CT-assisted navigation (BrainLab, Munich, Germany) and multimodal neuromonitoring (motor evoked potentials, somatosensory evoked potentials, and electromyography) were used to mitigate neurologic and vascular risk. Controlled derotation was performed by adjusting the Mayfield head holder, with the degree of correction limited by autofusion at C3-4. Fusion was augmented with local autograft and allograft.

This case highlights the complexity of operative planning and execution for progressive acquired cervical myelopathy in the setting of a very uncommon deformity with autofusion and aberrant vertebral artery anatomy. Navigation, neuromonitoring, and construct selection are useful risk-mitigation strategies when decompression and stabilization are required in high-risk anatomy.

## Introduction

Progressive cervical myelopathy results from sustained spinal cord compression and leads to gradual neurologic decline if left untreated. As the global population ages, the prevalence of degenerative spinal pathology and cervical myelopathy continues to rise, increasing the burden of complex spinal disorders encountered in clinical practice [1]. While most cases arise from symmetric degenerative changes, less common patterns of acquired deformity may present unique anatomic and surgical challenges.

Cervical spinal alignment plays a critical role in maintaining normal biomechanical load distribution and protecting neural elements. In the healthy cervical spine, axial loads are transmitted along the instantaneous axis of rotation and shared between the anterior and posterior spinal columns, allowing lordotic alignment to resist compressive forces efficiently [2]. Disruption of this balance alters load transmission across the cervical spine, predisposing to deformity progression and neural compression. Cervical kyphosis and scoliosis may arise from a variety of etiologies, including trauma, degenerative changes, prior surgery, or rheumatological conditions, with progression influenced by facet joint disruption, altered load transmission, and segmental autofusion [2,3].

Facet joints play a critical biomechanical role in guiding spinal motion and load sharing, and injury or degeneration of these structures can contribute to instability, deformity progression, and neurologic compromise [4]. Although degenerative and iatrogenic kyphosis are most common, trauma-related deformities may progress years after the inciting event, particularly when associated with facet joint disruption, altered load transmission, or progressive autofusion [3].

Unilateral facet pathology and asymmetric autofusion are uncommon but clinically significant, as they may produce rigid, rotational deformities with severe focal stenosis. In such cases, progressive myelopathy represents a clear indication for surgical decompression, as delayed intervention is associated with worse neurologic outcomes [4,5].

Surgical management of complex cervical deformity is further complicated by proximity to critical neurovascular structures, particularly the vertebral arteries. Anatomical variations in vertebral artery course are common and may include high-riding arteries, medial loops, or aberrant trajectories that place the vessel at increased risk during decompression or instrumentation [6-9]. Recognition of these variants through meticulous preoperative imaging is essential to mitigate the risk of catastrophic vascular injury, especially in deformity correction requiring multilevel fixation [6,7].

This case report describes a rare and complex presentation of progressive acquired cervical deformity with myelopathy in a 55-year-old woman, characterized by rigid kyphoscoliosis, autofusion at the C3-4 level, severe focal stenosis, and aberrant vertebral artery anatomy. The case highlights surgical decision-making and risk mitigation strategies necessary to safely manage atypical deformity patterns in patients with progressive cervical myelopathy.

## Case presentation

Patient information

A 55-year-old woman presented with progressive neurologic decline in the setting of an acquired cervical spine deformity. The patient recalled a fall approximately three years before presentation but did not undergo a comprehensive evaluation at that time. Over subsequent years, she developed progressive cervical deformity and myelopathic symptoms. Her medical history was notable for hepatic cirrhosis presumed secondary to alcohol use, complicated by portal hypertension requiring prior esophageal variceal banding and paracentesis for ascites. She had a history of multiple abdominal surgeries, including colonic resections related to prior iatrogenic perforation associated with toxic megacolon. Additional comorbidities included anxiety, depression, post-traumatic stress disorder, and attention-deficit/hyperactivity disorder. At the time of presentation, she was not using chronic opioid medications and reported minimal ongoing alcohol use.

Clinical findings

The patient presented with progressive cervical myelopathy, manifested by frequent falls, hand dysfunction, and sensory deficits. Neurologic examination demonstrated left biceps and triceps strength of 4/5, bilateral intrinsic hand and finger flexion strength of 2/5, and significant bilateral hand numbness.

Imaging studies

**Figure 1:**
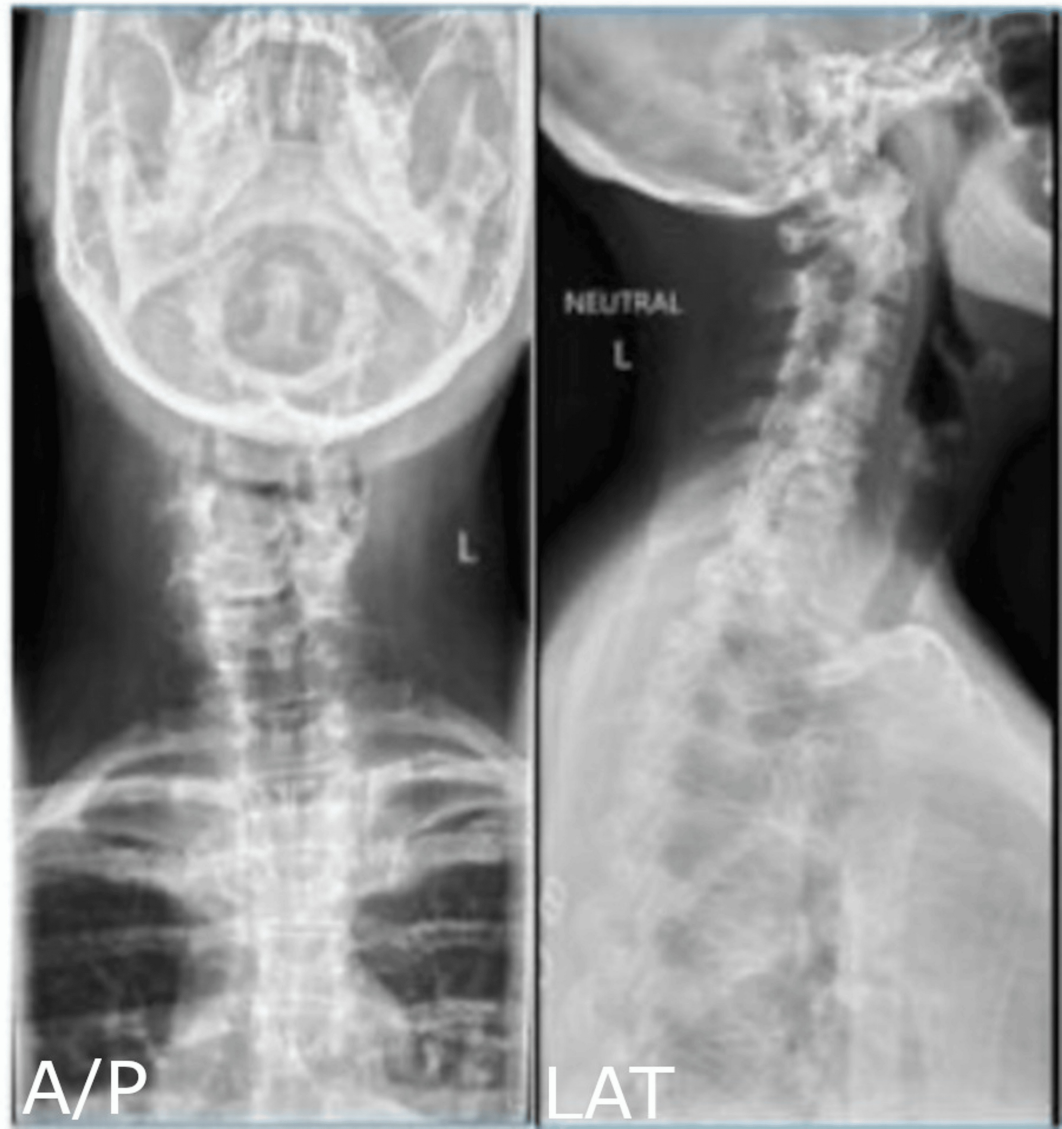
Initial Upright Cervical Radiographs Upright cervical radiographs obtained three years prior demonstrating loss of cervical lordosis, mid-cervical disc degeneration, mild upper cervical scoliosis, and grade I C4-5 spondylolisthesis. A/P: anteroposterior; LAT: lateral

**Figure 2:**
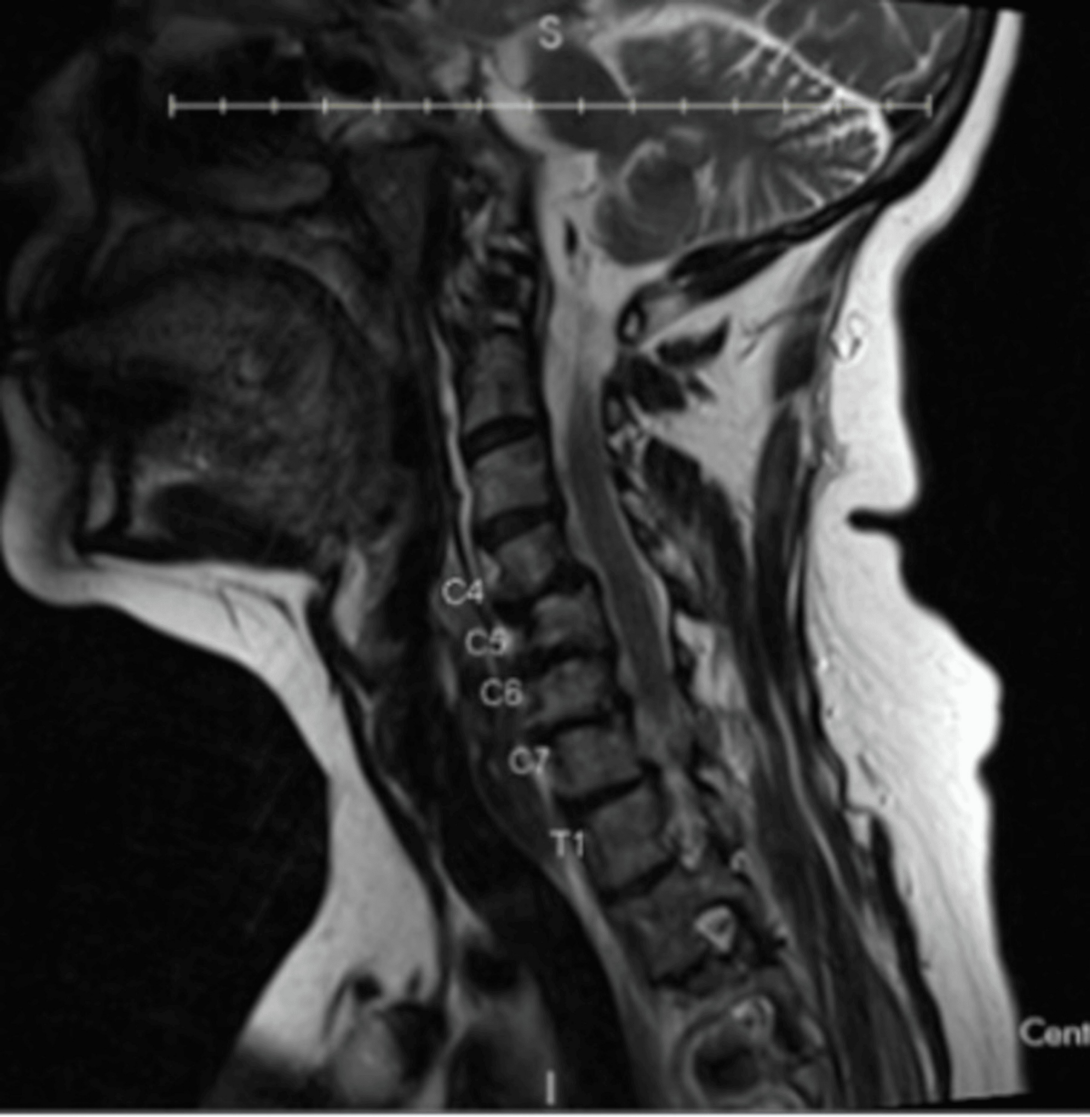
Initial Mid-sagittal T2-Weighted MRI MRI obtained three years prior demonstrating mild spinal canal stenosis and C4-5 spondylolisthesis without facet dislocation.

Serial imaging demonstrated progression from mild deformity to a rigid cervical kyphoscoliosis. Earlier cervical radiographs obtained three years prior revealed minimal upper cervical scoliosis and grade I C4-5 spondylolisthesis with preserved overall alignment (Figure 1). Cervical magnetic resonance imaging (MRI) at that time demonstrated mild canal narrowing as well as the C4-5 spondylolisthesis (Figure 2).

**Figure 3:**
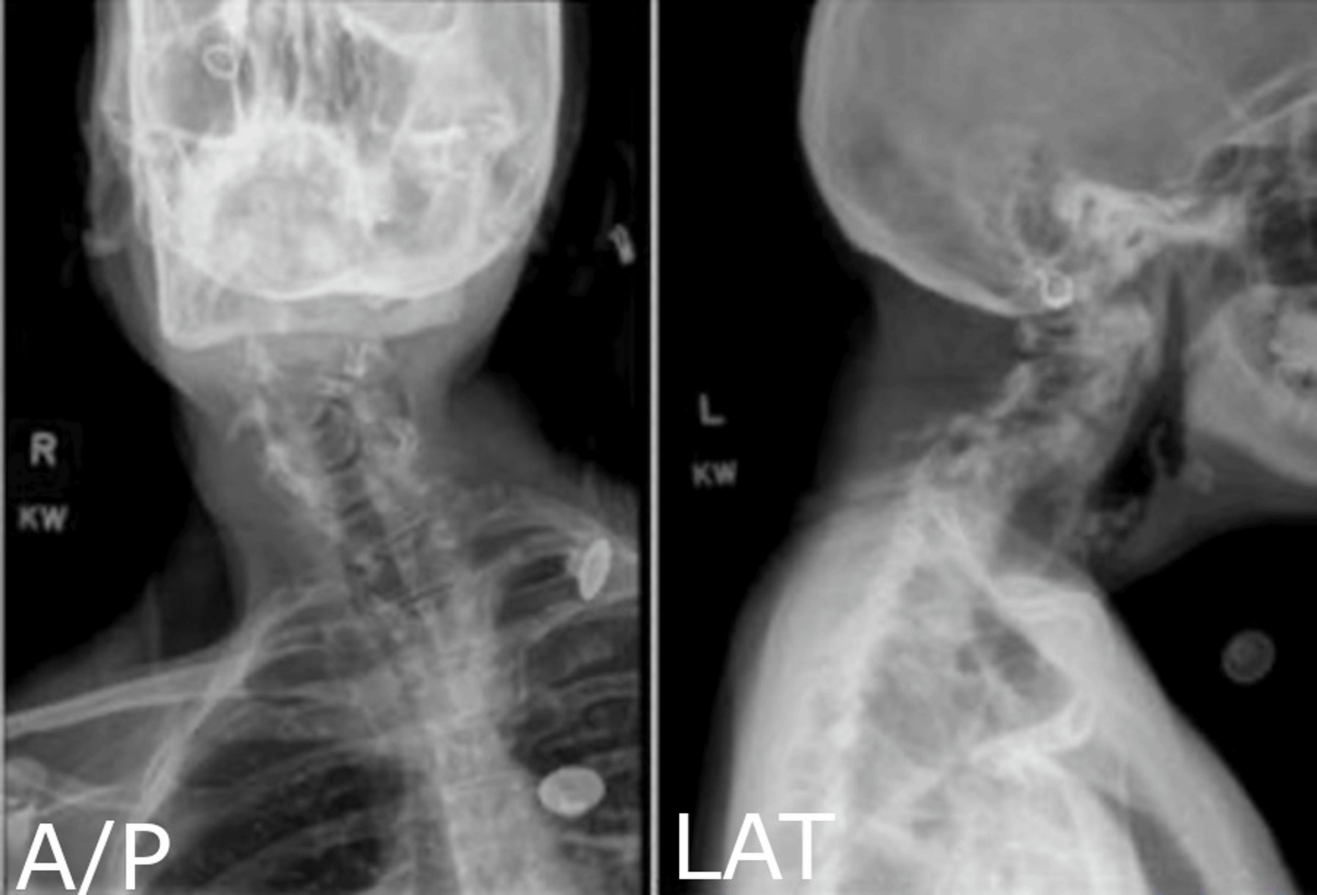
Preoperative Upright Cervical Radiographs Preoperative cervical radiographs demonstrating rigid apex-right scoliosis with a Cobb angle of 27 degrees, segmental kyphosis, C4-5 spondylolisthesis, with no deformity correction on supine A/P X-ray with axial traction. K Line (not shown) is negative. A/P: anteroposterior; LAT: lateral

Preoperation upright cervical radiographs demonstrated rigid apex-right scoliosis (Cobb angle 27 degrees), segmental kyphosis, C4-5 spondylolisthesis, with no deformity correction on supine anteroposterior (AP) X-ray with axial traction (Figure 3).

**Figure 4:**
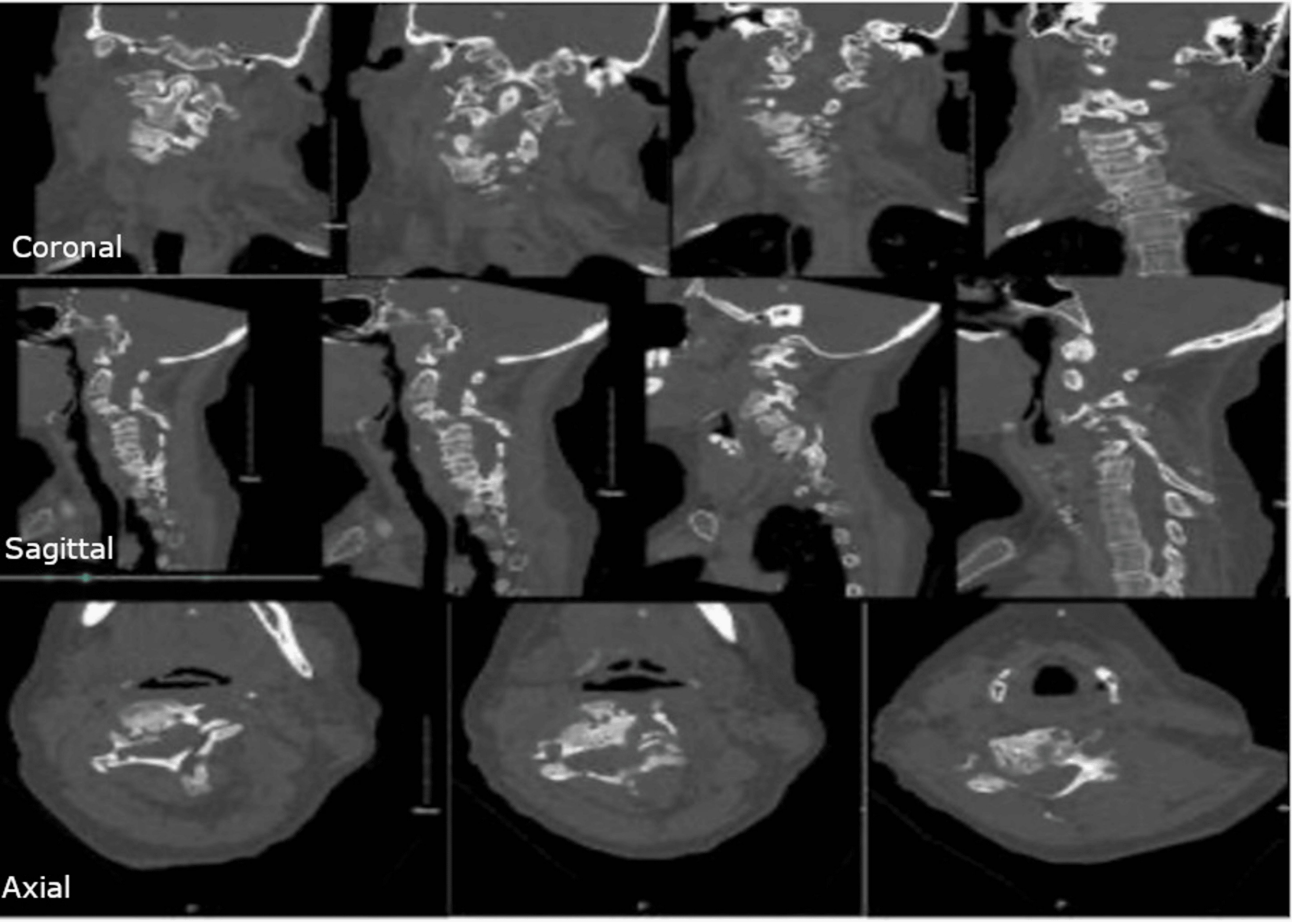
Preoperative Cervical CT Preoperative cervical CT images (sagittal, coronal, axial) demonstrating left-sided facet dislocation at C4-5, autofusion across the C3-4 disc space and right facet joint, asymmetric pedicle bone loss, and rigid cervical kyphoscoliosis.

Computed tomography (CT) revealed thin laminae at C3-5 on the left, autofusion across the C3-4 disc space and right facet joint, a unilateral left-sided facet dislocation at C4-5, and extensive asymmetric bony loss involving the left pedicle and lateral mass at C4-5 (Figure 4).

**Figure 5:**
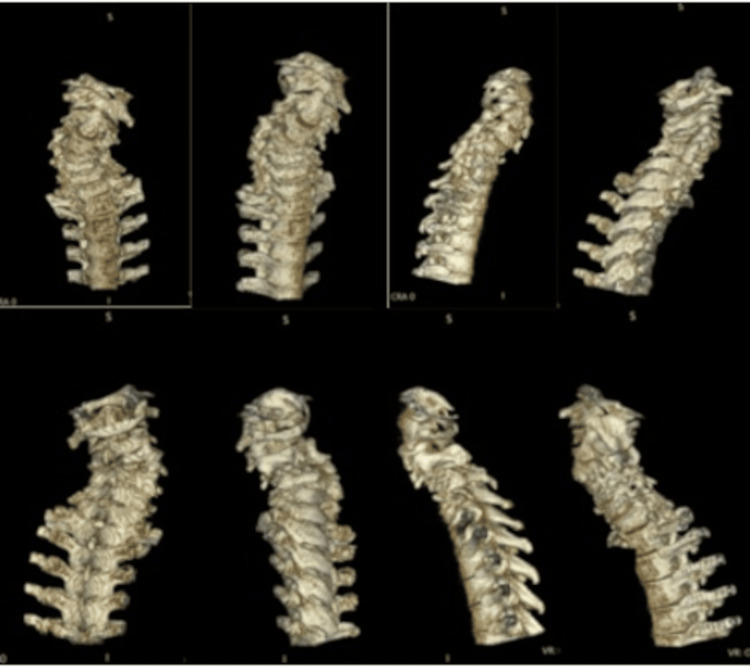
Three-Dimensional Reconstructed Cervical CT A 3D-reconstructed cervical CT was performed to visualize better the extent of the rotational component of the deformity.

Three-dimensional reconstructed CT further demonstrated severe deformity of the left C3 and C4 lateral masses contributing to rotational and rigid deformity (Figure 5).

**Figure 6:**
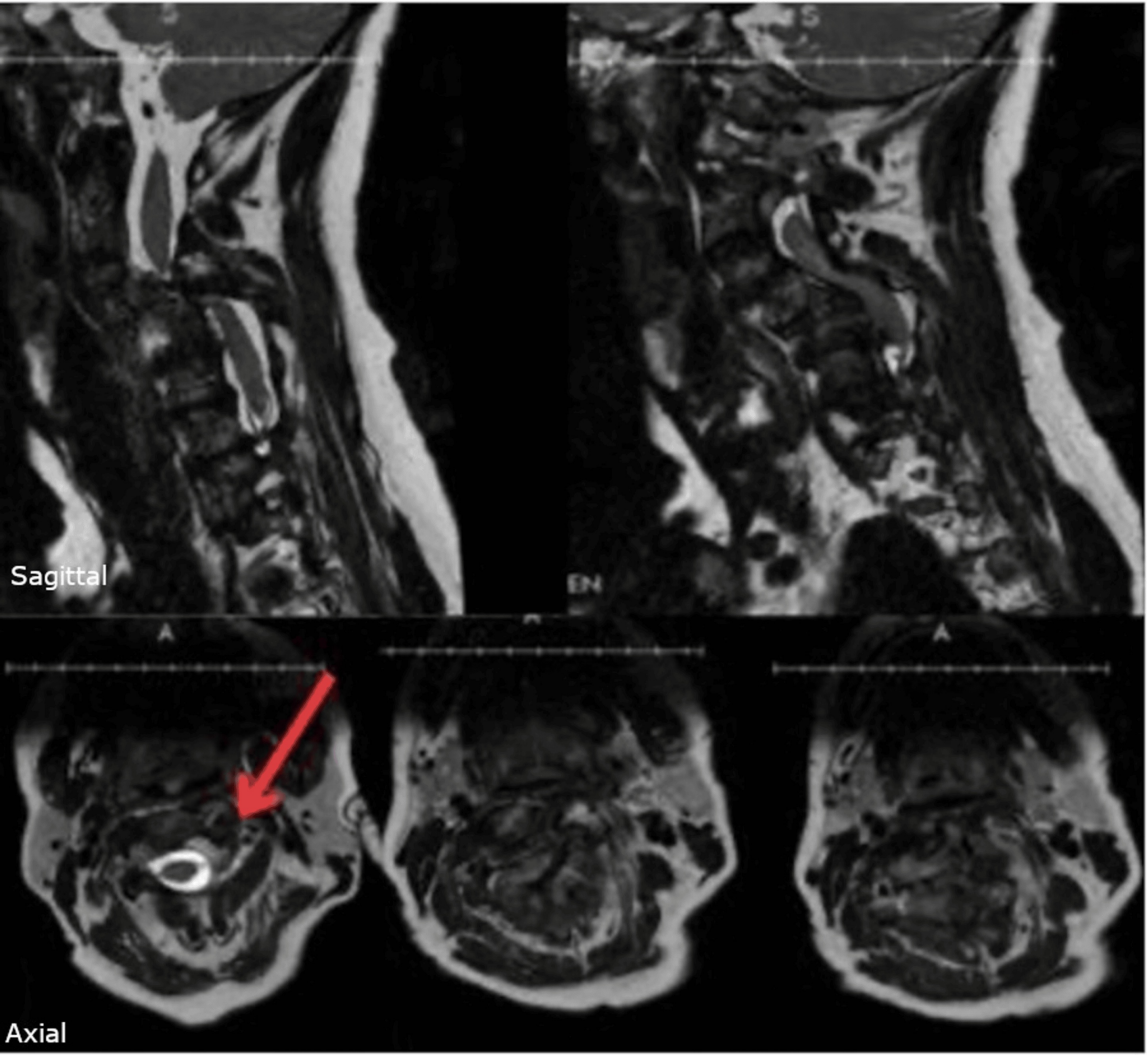
Preoperative T2-weighted Sagittal and Axial Cervical MRI Preoperative T2-weighted cervical MRI demonstrating severe multilevel spinal canal stenosis from C4-6 at the apex of cervical kyphosis and atypical vertebral artery anatomy on left at C2 (arrow).

Magnetic resonance imaging demonstrated severe focal spinal canal stenosis from C4-6 at the apex of cervical kyphosis, with associated spondylolisthesis at C3-4 and C4-5. Preoperative MRI and CT revealed atypical vertebral artery anatomy outside the vertebral foramen from C2-C4 on the left, significantly increasing the risk associated with lateral mass screw placement at those levels (Figure 6).

Management considerations

The patient had failed conservative management, including physical therapy and pharmacologic treatment. Given progressive neurologic decline, severe focal cord compression, and rigid deformity with autofusion, surgical intervention was recommended. Surgical options included anterior, posterior, and combined anterior-posterior approaches. Although multilevel anterior cervical discectomy and fusion (ACDF) or corpectomy is often considered the preferred method of cord decompression in the setting of kyphosis and a negative K line, this patient's atypical vertebral body morphology and vertebral artery anatomy, the number of levels involved, and the favorable C2-7 K-line, due to upper cervical lordosis and lower cervical kyphosis, led the surgeon to select a posterior approach. The patient was counseled extensively regarding the high-risk nature of the procedure, including the potential for intraoperative or postoperative neurologic deterioration and vascular injury related to aberrant vertebral artery anatomy.

Procedure

**Figure 7:**
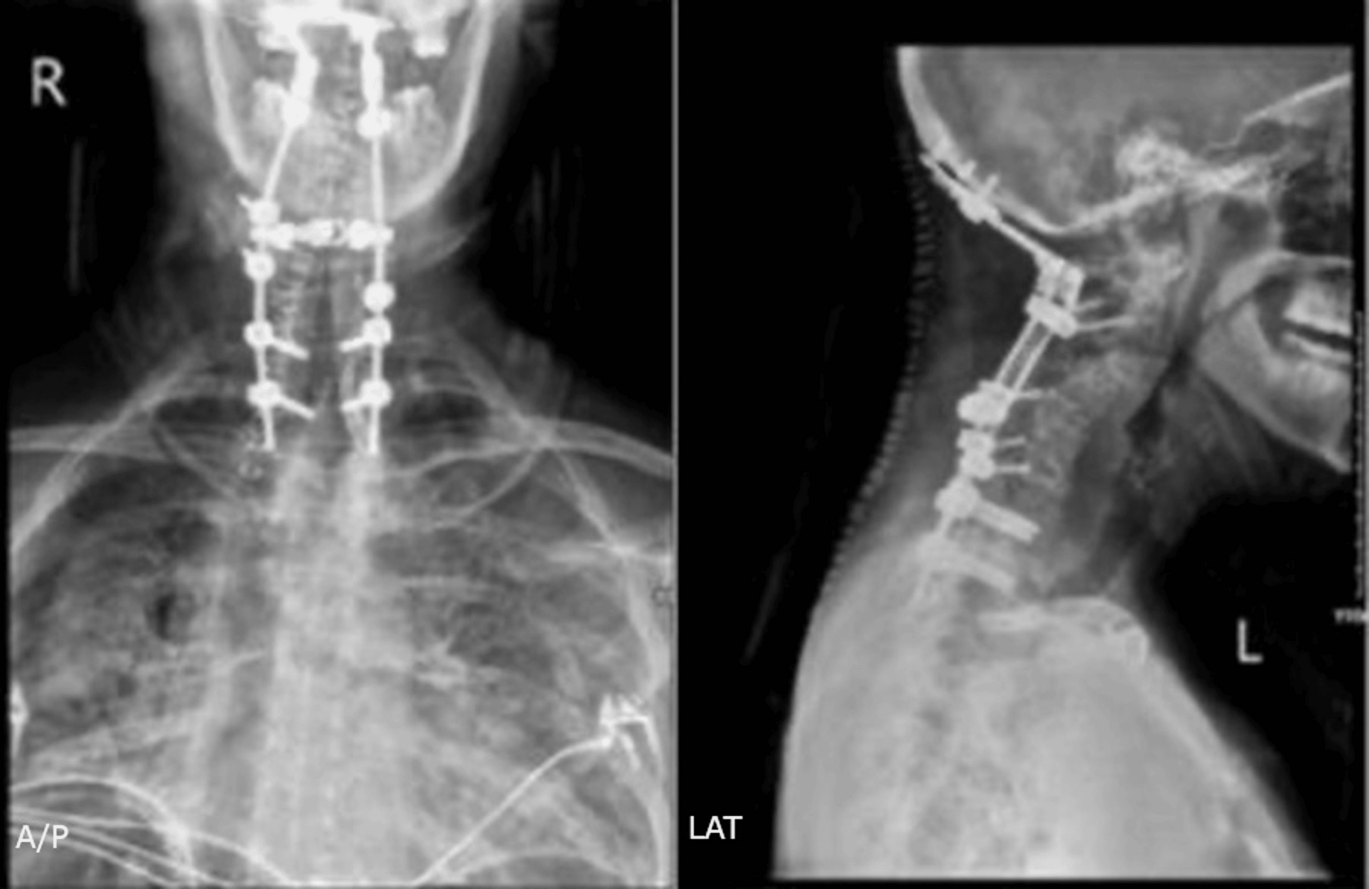
Postoperative Upright Cervical Radiographs Postoperative upright cervical radiographs demonstrating posterior occipitocervicothoracic instrumented fusion with partial deformity correction. A/P: anteroposterior; LAT: lateral

The patient underwent posterior cervical decompression and stabilization consisting of a C3-7 laminectomy and occiput to T2 instrumented fusion. Instrumentation included an occipital plate, C2 pars screws, subaxial lateral mass screws (with several levels unable to accept screws due to atypical bony morphology and vascular considerations), and T1-T2 pedicle screws using the DePuy-Synthes Symphony (Johnson & Johnson MedTech, Warsaw, USA) posterior cervical system (Figure 7).

Computed tomography-assisted navigation (BrainLab, Munich, Germany) and multimodal neuromonitoring, including motor evoked potentials, somatosensory evoked potentials, and electromyography, were utilized throughout the procedure. Following screw placement and laminectomy, facet joints were released, and controlled derotation was performed by adjusting the Mayfield head holder before securing the occipital plate and rods. Complete deformity correction was limited by autofusion across the disc space and right facet joint at C3-4. Local bone autograft and PliaFx (LifeNet Health, Virginia Beach, USA) allograft were placed to promote fusion. Total operative time was approximately four hours, with an estimated blood loss of 300 mL. No intraoperative complications occurred. A rigid cervical collar was applied postoperatively.

Follow-up and outcomes

The patient demonstrated immediate postoperative improvement in upper extremity strength and hand sensation. On postoperative day two, she developed acute shortness of breath secondary to significant abdominal distension from ileus and acute colonic pseudo-obstruction (Ogilvie syndrome), likely related to her extensive prior abdominal surgical history. She required temporary intubation and mechanical ventilation, with resolution following nasogastric decompression and therapeutic sigmoidoscopy. After extubation, neurologic examination confirmed sustained improvement in strength and sensation. Approximately three weeks postoperatively, she developed a minimally symptomatic pulmonary embolism while receiving prophylactic anticoagulation (enoxaparin) and was transitioned to therapeutic apixaban.

At the 4-week postoperative visit, her strength and sensation were normal in the upper and lower extremities with improvement in hand dexterity. At the 6-week postoperative follow-up, the patient demonstrated appropriate wound healing of the head, neck, and posterior cervical incision sites. Neurologic examination revealed 5/5 strength bilaterally in the deltoids, biceps, triceps, wrist extensors, finger flexors, and intrinsic hand muscles, with intact sensation to light touch in the C5-T1 dermatomal distributions. She was advised to continue wearing a cervical collar and to maintain bending, lifting, and twisting restrictions until her next evaluation. Plans were made for a 9-week postoperative follow-up with two-view cervical radiographs. At this visit, the patient was successfully weaning off narcotic pain medications and was provided a prescription for outpatient physical therapy.

## Discussion

Progressive acquired cervical deformity with myelopathy represents a challenging clinical entity, particularly when deformity becomes rigid and is compounded by autofusion and aberrant vascular anatomy. In this case, delayed progression following a suspected remote traumatic event likely contributed to asymmetric facet disruption, altered load transmission, and progressive kyphoscoliosis, ultimately resulting in severe focal spinal canal stenosis and neurologic compromise.

Rigid deformity with autofusion significantly limits the utility of traction and restricts the degree of safe deformity correction. Additionally, asymmetric lateral mass and pedicle morphology, combined with anomalous vertebral artery anatomy, markedly increase the technical complexity and vascular risk associated with cervical instrumentation. In such cases, meticulous preoperative imaging, CT-based navigation, and neuromonitoring are helpful to mitigate neurologic and vascular injury.

The patient’s early postoperative complications highlight the need for multidisciplinary perioperative management, particularly in patients with significant medical comorbidities. However, her sustained neurologic improvement illustrates that, in carefully selected patients, timely surgical decompression can yield meaningful functional recovery despite substantial operative risk.

## Conclusions

This case illustrates the complexity of diagnosis and surgical management of progressive acquired cervical myelopathy resulting from severe left C4-5 facet dislocation with associated spondylolisthesis and segmental autofusion at C3-4, leading to rigid focal deformity and severe spinal canal stenosis. The unilateral nature of the osseous deformity at C3-4 highlights how focal lateral asymmetric load transmission can drive deformity progression and neurologic compromise, even in the absence of a clearly defined acute traumatic event. Successful management required careful preoperative planning, intraoperative navigation, and acceptance of partial deformity correction to prioritize neurologic safety.
